# Organ-Specific Extracellular Vesicles in the Treatment of Ischemic Acute Organ Injury: Mechanisms, Successes, and Prospects

**DOI:** 10.3390/ijms26199709

**Published:** 2025-10-06

**Authors:** Irina B. Pevzner, Nadezda V. Andrianova, Anna K. Lomakina, Kseniia S. Cherkesova, Elizaveta D. Semenchenko, Egor Y. Plotnikov

**Affiliations:** 1 A.N. Belozersky Institute of Physical-Chemical Biology, M.V. Lomonosov Moscow State University, 119991 Moscow, Russia; andrianova@belozersky.msu.ru (N.V.A.);; 2 Faculty of Bioengineering and Bioinformatics, M.V. Lomonosov Moscow State University, 119991 Moscow, Russia; 3 Biological Faculty, M.V. Lomonosov Moscow State University, 119991 Moscow, Russia

**Keywords:** extracellular vesicles, regenerative medicine, neuroinflammation, apoptosis, angiogenesis, kidney, brain, heart

## Abstract

Ischemia–reperfusion (I/R) injury is a complex pathological process underlying numerous acute organ failures and is a significant cause of morbidity and mortality in diseases such as myocardial infarction, stroke, thrombosis, and organ transplantation. Mesenchymal stem cell (MSC)-derived extracellular vesicles (EVs) have demonstrated considerable therapeutic potential, but their broad tropism and general repair signaling may limit their efficacy. This review addresses the emerging paradigm of using organ-specific EVs for the treatment of I/R injury in the respective organs. We summarize the existing studies performed on experimental animals showing that these native EVs could possess tissue tropism and carry a specialized cargo of proteins, miRNAs, and lipids tailored to the unique regenerative needs of their organ of origin, enabling them to precisely modulate key processes, including inflammation, apoptosis, oxidative stress, and angiogenesis. However, their clinical translation faces challenges related to scalable production, standardization, and the dualistic nature of their effects, which can be either protective or detrimental, depending on the cellular source and pathophysiological context. Future developments need to focus on overcoming these obstacles through rigorous isolation protocols, engineering strategies such as cargo enrichment and hybrid vesicle creation, and validation in large-animal models. Overall, organ-specific EVs offer a novel, cell-free therapeutic strategy with the potential to significantly improve outcomes in I/R injury.

## 1. Introduction

Ischemic injury, caused by an impairment of blood supply to tissues, results in a critical lack of oxygen and nutrients, ultimately causing cellular dysfunction and death [1]. The subsequent restoration of blood supply, known as reperfusion, is necessary to save the tissue but can paradoxically exacerbate the damage through a cascade of events known as ischemia/reperfusion (I/R) injury [2]. This phenomenon is characterized by a burst of reactive oxygen species (ROS), intracellular calcium overload, profound inflammation, and the activation of programmed cell death pathways [1]. Ischemic damage to the brain, heart, kidneys, liver, lungs, and other organs can lead to serious disorders, including myocardial infarction (MI), stroke, solid organ transplantation, and acute kidney injury (AKI) [3,4,5,6]. I/R injury to a single organ can trigger a subsequent inflammatory response in remote organs. It is well-established that ischemic stroke can lead to AKI, while hepatic I/R injury can induce intestinal inflammation, ultimately progressing to multiple organ dysfunction syndrome [1,7,8]. Therefore, the development of effective therapeutic strategies for I/R remains an urgent problem in modern medicine.

In recent years, extracellular vesicles (EVs) have emerged as a promising next-generation therapeutic approach for mitigating I/R injury [9]. EVs are nanoscale particles with a lipid bilayer that are secreted by all cell types [10]. They act as crucial mediators of intercellular communication by transporting a diverse cargo of bioactive macromolecules, including proteins, lipids, mRNAs, and microRNAs (miRNAs), from producing cells to recipient cells [10]. Their small size (typically 30–150 nm for exosomes and 100–1000 nm for microvesicles) enables them to circulate and navigate in the blood and pass through biological barriers, facilitating different signals [10,11]. In this review, we use the term “EVs” as a general descriptor unless the original study specifically characterizes exosomes or microvesicles.

The protective effects of EVs in the I/R context are diverse and are primarily attributed to the functional delivery of their molecular cargo [12]. Due to their advantageous properties—including stability, immune tolerance, biocompatibility, non-toxicity, ability to cross biological barriers, and inability to replicate—EVs represent a promising therapeutic platform [13]. Their mechanisms of action include potent anti-inflammatory effects and the establishment of a durable immune response [4,11,13,14]; powerful anti-apoptotic signals via the transfer of pro-survival miRNAs and the activation of pro-survival signaling pathways like PI3K (phosphatidylinositol-3 kinase)/Akt (protein kinase B) [15]; the promotion of angiogenesis and cell proliferation by delivering pro-angiogenic factors and miRNAs [16,17]; and the reduction in oxidative stress through the transfer of antioxidant enzymes [13,18,19,20]. This multi-modal approach allows EVs to effectively counteract the key pathological processes driving I/R damage.

While mesenchymal stem cell (MSC)-derived EVs have been extensively studied and demonstrate significant efficacy, their application may have limitations, such as the lack of innate tissue-specific targeting, low payload, and functional dependence on the microenvironment [20,21,22]. An alternative is to use EVs derived directly from the organ susceptible to injury, for instance, cardiac-derived EVs to treat MI or kidney-derived EVs to treat AKI [13,23,24]. These organ-specific EVs are thought to possess a natural tropism for their tissue of origin and carry a specialized cargo tailored to the particular regenerative needs of the damaged organ, potentially offering superior targeting and efficacy compared to their MSC-derived counterparts.

**Figure 1:**
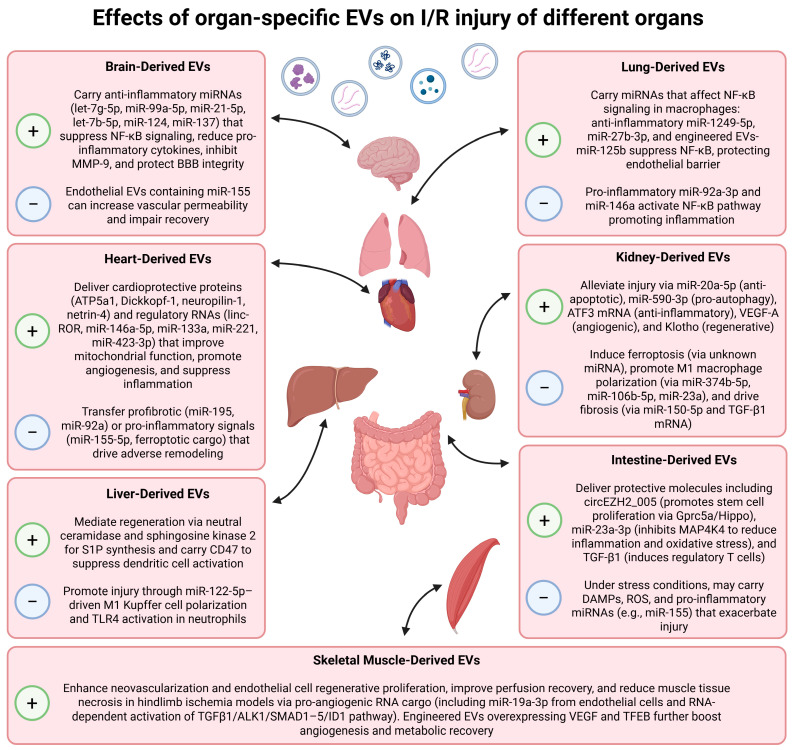
The role of organ-specific EVs in modulating I/R injury in different organs. This schematic summarizes the dualistic effects of EVs from specific organ cells on their native tissue following I/R damage. The outcome, whether protective or detrimental, is highly dependent on the molecular cargo of EVs, the physiological state of the parent cell, and the timing of administration after injury.

Therefore, the aim of this review was to summarize and evaluate the current evidence on the application of organ-specific EVs for the treatment of I/R injury in the respective tissues. We compiled preclinical results, discussed proposed mechanisms of action, and assessed the potential advantages and challenges of this emerging targeted therapeutic approach, as well as considered some limitations of their use. Next, we will describe organ-specific EVs and possible mechanisms of their action in ischemic pathologies (Figure 1).

## 2. Heart-Derived EVs

MI remains a leading cause of mortality worldwide, underscoring the critical need for novel therapeutic strategies. A key mechanism of injury in MI is ischemia followed by reperfusion, which triggers massive cardiomyocyte death through apoptosis and necrosis, activates detrimental inflammatory responses, and promotes pathological cardiac remodeling. It was found, in response to ischemic injury, that cardiac cells could produce EVs that participate in tissue reaction to pathological processes. Although the precise role of endogenous EVs in MI (whether protective or detrimental) and molecular mechanisms of action are not fully understood, this phenomenon suggests a promising therapeutic opportunity. EVs released by damaged myocardium may exacerbate injury to surrounding cells. Consequently, the administration of EVs obtained from healthy cardiac cells in vitro has been proposed as a strategy for targeted delivery of protective signals during MI. This hypothesis has motivated investigations into specific EVs populations derived from cardiomyocytes, fibroblasts, or cardiac endothelial cells to treat MI by targeting key injury mechanisms.

### 2.1. Cardiomyocyte-Derived EVs

Cardiomyocyte-derived EVs produced after MI participate in cardiac remodeling by mediating paracrine interactions between cardiomyocytes, fibroblasts, and endothelial cells [25]. Recent studies have shown that cardiomyocyte-derived exosomes have many pivotal biological functions, like influencing the progress of coronary artery disease via modulating macrophage phenotypes [26]. EVs isolated from the ventricular cardiomyocyte cell line AC10 grown under hypoxia promoted angiogenesis and fibroblast cell motility in the scratch assay in vitro. Mass spectrometry showed that only EVs produced by cardiomyocytes after hypoxia, but not after normoxia, contained cardiogenic and cardioprotective proteins (Dickkopf-related protein 1, neuropilin 1, and netrin 4) and proteins related to cellular stress response (ATP-citrate synthase, fatty acid synthase, X-ray repair cross-complementary protein 5, and aminopeptidase N) [26].

The interaction of EVs and recipient heart cells could be mediated by connexin 43 (Cx43), and a decrease in the content of Cx43 in EVs during MI could be one of the mechanisms of disruption of intercellular communication, but after 4 h of reperfusion, the level of Cx43 in EVs was restored. Cx43 is ubiquitinated in EVs, and ischemia can stimulate degradation of ubiquitinated Cx43 rather than suppress EV production. The lack of ATP and/or oxygen during ischemia may be the cause of changes in the activation of signaling pathways, and the p62 autophagy adapter may act as a putative regulator of the balance between Cx43 degradation and secretion [27].

Cardiomyocyte-derived EVs could mediate the activation of cardiac fibroblasts (CFs) and lead to myofibroblast phenotype conversion, which can contribute to heart recovery after MI. As has been shown in a murine model of MI, the effect can be achieved due to overexpression of miR-195 after ischemia, since miR-195 targets the SMAD (small mother against decapentaplegic) 7, which inhibits expression of α-SMA (alpha-smooth muscle actin), a marker of myofibroblast activation [28]. It has been reported that SMAD7 is a target of miR-92a, whose levels were significantly increased in cardiomyocyte-derived exosomes and in fibroblasts isolated after MI. Exosomes transfer miR-92a to CF after MI, where miR-92a suppresses SMAD7 transcription and activates the conversion of fibroblasts into myofibroblasts [29].

EVs derived in vitro from the murine cardiomyocyte cell line HL1 increased endothelial cell proliferation by 25% and migration by 50%. This may be due to a change in the expression pattern of a number of matrix metalloproteases in endothelial cells after treatment with EVs, namely, an increase in the mRNA and protein levels of matrix metalloproteinase (MMP)-3, and a significant decrease in the expression of *Mmp-2* and *Mmp-10 genes*. At the same time, the treated endothelial cells lost their ability to form tube-like structures with other cells, which may be due to stimulation of the p53 and p16 expression—that is, cardiomyocyte-derived EVs can induce apoptosis and senescence of endothelial cells. Although HL1-derived EVs enhanced endothelial migration in vitro, their stability, biodistribution, and functional activity in the complex post-MI microenvironment remain to be validated in large-animal models [30]. The effect of cardiomyocyte-derived EVs on the cardiac vascular endothelial cells can be mediated by long non-coding RNAs. The transfer of long intergenic non-protein coding RNA (linc-ROR) by EVs into the cardiac microvascular endothelial cells reduced the levels of miR-145-5p and, thereby, increased the expression of p70s6k1 (Ribosomal protein S6 kinase beta-1) and enhanced its phosphorylation, which led to activation of the eNOS (endothelial–nitric oxide synthase) pathway, improving microcirculation and reducing damage to cardiac tissues. The effect depended on the EV transfer of linc-ROR and was abolished if cardiomyocytes were preliminarily treated with GW4869, which blocks the generation of EVs [31].

It is well-known that macrophage polarization into the pro-inflammatory M1 phenotype may promote an inflammatory response and increase myocardial damage in MI. Incubation of RAW264.7 macrophages with exosomes from neonatal mouse cardiomyocytes overexpressing miR-146a-5p modulated M1 macrophage polarization, reducing the level of pro-inflammatory cytokines (TNFa (tumor necrosis factor alpha) and iNOS (inducible inducible nitric oxide synthase)) and the NF-κB (nuclear factor κB) signaling pathway factors (TRAF6 (TNF receptor associated factor 6) and its downstream target phosphorylated protein IKK (IkappaB kinase) a/β) [32]. On the contrary, miR-155-5p, transferred to macrophages as cargo of cardiomyocyte-derived EVs, can act as a pro-inflammatory agent and contribute to M1 macrophage polarization in a murine model of MI by activation of the JAK2 (Janus kinase)/STAT1 (signal transducer and activator of transcription) pathway [33]. The M1 macrophage polarization can also occur as a result of the activation of Wnt/β-catenin signaling. Treatment of RAW 264.7 macrophages with exosomes secreted by ferroptosis-undergoing cardiomyocytes in MI models of HL-1 cells induced by hypoxia increased the expression of Wnt1 and β-catenin and led to M1-polarization, and preliminary treatment of cardiomyocytes with the ferroptosis inhibitor ferrostatin-1 decreased M1 polarization of RAW 264.7 [34].

EVs from the cardiac tissues of newborn mice, intact or exposed to ventricular apex resection, when administered into the peri-infarct area of the heart, possessed a significant cardioprotective effect, stimulating angiogenesis and the proliferation of mono-nucleated cardiomyocytes. However, no therapeutic effect was observed after treatment with EVs derived from the hearts of adult mice. The beneficial effect of the EVs could be associated with an increase in Wdr75 expression in cardiomyocytes after ventricular apex resection and associated suppression of p53 protein expression in cardiomyocytes and endothelial cells [35].

Intramyocardial administration of cardiomyocyte-derived EVs from healthy heart tissue improved cardiac function and caused remodeling of the heart, and this effect was mediated through delivery of ATP5a1 and restoration of energy metabolism of mitochondria. Incubation of cardiomyocytes subjected to hypoxia/reoxygenation with these EVs suppressed ROS production in mitochondria and caused resistance to damage caused by oxidative stress [36].

### 2.2. Cardiac Fibroblasts-Derived EVs

CFs are able to protect cardiomyocytes from lethal damage under hypoxia/reoxygenation conditions in vitro; moreover, specifically CF secretome is responsible for the cardioprotective effect. EVs derived from CFs (CF-EVs) were enriched in the TIMP-1 (tissue inhibitor of metalloproteinase-1). These EVs reduced cell necrosis and apoptosis in vitro, and the protective effect was abolished by TIMP-1 depletion. Intravenous administration of CF secretome and independently TIMP-1 before reperfusion reduced infarct size of the left ventricle. The cardioprotective mechanism is suggested to be associated with the activation of PI3K/Akt and ERK (extracellular-signal-regulated kinase) 1/2 signaling pathways by TIMP-1 [37].

It is reported that a number of miRNAs in CF-EVs can have a cardioprotective effect. GATA4+ exosomes derived from cardiac colony-forming unit fibroblasts exhibited cardioprotective effects in vivo and in vitro due to antiapoptotic miR221, which was overexpressed in GATA4+ exosomes. Targeting miR221 to the PTEN (phosphatase and tensin homolog) target gene led to a decrease in PTEN protein levels, activated the PI3K/Akt signaling pathway, and subsequently reduced apoptosis of myocardial cells [38]. miR-133a, which is overexpressed in CF under hypoxia/reoxygenation, also exhibits cardioprotective properties. miR-133a can target 3′-UTR ELAVL1 (embryonic lethal vision-like protein 1) and suppress its expression, thereby suppressing pyroptosis of cardiomyocytes in vitro. In a rat model, intravenous administration of exosomes derived from CF overexpressing miR-133a reduced the levels of pyroptosis markers ELAVL1, NLRP3 (nucleotide-binding domain, leucine-rich repeat, and pyrin domains-containing protein 3), and caspase-1 in cardiac tissues and the levels of inflammatory cytokines IL (Interleukin)-1beta and IL-18 in blood [39]. Exosomes enriched with miR-423-3p mediated the protective effect of CF on H9C2 cardiomyocytes when co-cultured under hypoxia/reoxygenation conditions in vitro. Elevated levels of miR-423-3p suppressed RAP2C (Ras-related protein 2C) expression, which reduced apoptosis in H9C2 cells [40].

### 2.3. Endothelial-Cell-Derived EVs

Endothelial-cell-derived EVs can exhibit a protective effect through activation of the MAPK (mitogen-activated protein kinase) ERK1/2 signaling pathway. In a study by Davidson et al., the preliminary treatment of cardiomyocytes with two inhibitors of the MEK1/2 (mitogen-activated protein kinase kinases 1 and 2)–ERK1/2 kinase pathway abolished cardioprotection by exosomes derived from the HUVEC endothelial cell line [41]. It was shown that endothelial-cell-derived EVs were enriched with MEK1/2 and HSP90 (heat shock protein 90), which may be responsible for the cardioprotective effect through the MEK1/2/eNOS/guanylyl cyclase pathway, while the inflammatory microenvironment impaired the cardioprotective effect of EVs. EVs from endothelial cells preliminarily treated with IL-3 were enriched with the eNOS-antagonist caveolin-1 (Cav-1) and proteins associated with the inflammatory response and did not show a cardioprotective effect on the H9c2 cardiomyocyte hypoxia/reoxygenation model and the rat I/R model [42].

Endothelial-cell-derived EVs are able to reduce intramuscular myocardial damage in MI due to their miRNA cargo. Thus, miR-24-3p miRNA in EVs isolated from endothelial cells overexpressing KLF2 (Kruppel-like factor 2) inhibited the migration of pro-inflammatory monocytes with high Ly6C expression levels from bone marrow to damaged cardiac tissues in vivo. miR-24-3p suppressed the expression of CCR2 (CC-chemokine receptor 2), a receptor needed for cell migration, which resulted in reduced levels of inflammation in the heart and diminished myocardial damage [43]. Shock wave therapy of an ischemic heart caused the release of exosomes enriched in miR-19a-3p from endothelial cells. Such exosomes exhibited angiogenic properties by activating Akt and ERK, which led to increased endothelial tube formation and proliferation [44]. Akbar et al. [45] revealed that peripheral blood EVs are enriched in a number of miRNAs in patients with MI, including miR-632, miR-126-3p, miR-151a-3p, miR-26b-5p, miR-126-5p, let-7a-5p, miR-1972, miR-15b-5p, miR-23a-3p, miR-374b-5p, miR-23b-3p, and let-7b-5. miR-126-3p and miR-126-5p accumulated in the spleen of healthy rats and mobilized spleen monocytes. A possible mechanism is increased expression of genes associated with chemotaxis, including ITGA4 (integrin subunit alpha 4) and ITGB2 (integrin subunit beta 2), subunits of the integrin VLA-4 (very late antigen-4), a receptor for the VCAM-1 (vascular cell adhesion molecule 1) protein. VCAM-1 is overexpressed in endothelial-cell-derived EVs and is involved in the recognition and binding of EVs to monocytes, providing their activation and mobilization after MI, which aggravates inflammation and impairs cardiac function.

### 2.4. Advantages over MSC-EVs

EVs derived from cardiac tissues have several advantages in the treatment of heart pathologies over EVs obtained from MSCs. First, they carry cargo specific to cardiac tissues. The results of miRNA sequencing and DESeq2 analysis showed that 219 miRNAs are expressed differentially in EVs from cardiomyocytes versus MSC-EVs, and the differential expression of these miRNAs was confirmed by RT-qPCR. Mapping the targets of differentially expressed miRNAs revealed that the pathways of differentially expressed miRNAs are involved in heart-specific processes [46].

Secondly, EVs from cardiac tissues are not arrhythmogenic and promote recovery of contractile function. In a rat model of MI, animals with ventricular arrhythmias were injected with CM-EVs and experienced significantly fewer arrhythmic events [46]. Echocardiograms showed that CM-EVs treatment increased the ejection fraction and reduced cardiac dilation 2 weeks after induction of MI, and after 4 weeks, there were no significant differences with control animals in diastolic diameter, systolic diameter, and ejection fraction [46].

Thirdly, organ-specific EVs are able to specifically target cardiac tissues and can be used as vectors for targeted delivery. Cargo of conventional delivery systems (lipid nanoparticles or stem cell-derived EVs) tends to accumulate in the liver, which is why therapeutic molecules do not reach the intended organ. In a study by Nawaz et al., VEGF-A (vascular endothelial growth factor A) mRNA was delivered to the mice’s hearts by using EVs derived from cardiac progenitor cells (CPC-EVs). EVs were administered intravenously, and only CPC-EVs, compared with lipid nanoparticles and stem cell-derived EVs, showed an efficient targeted delivery of the VEGF-A mRNA to the heart tissue. Direct injection of EVs and lipid nanoparticles carrying an equal amount of VEGF-A mRNA into the myocardium also showed that CPC-EVs induced higher production of VEGF protein compared with other delivery vehicles or the administration of naked mRNA. Moreover, the VEGF-A protein was not localized in the nearby regions of the heart, liver, or blood, which indicates the absence of off-target and effective absorption of EVs cargo by heart tissues [47].

Fourth, organ-specific EVs exhibit lower immunogenicity compared to EVs from stem cells and lipid nanoparticles. VEGF-A mRNA delivery using CPC-EVs caused the least changes in gene expression in cardiac tissue (only 82 transcripts were deregulated against 437, 445, and 240 when using lipid nanoparticles, EVs derived from a human lung epithelial cell line, and EVs derived from human umbilical vein endothelial cells, respectively). A principal component analysis showed significant differences in the transcriptomic profiles of the groups injected with three types of EVs and lipid nanoparticles, naked VEGF-A RNA, and the control group (37% of the variance), and only upon delivery of CPC-EV-activated genes were they associated with “cardiac muscle cell development” and “factors that promote cardiogenesis in vertebrates”. CPC-EV did not induce upregulation of inflammation genes in the heart, while other delivery vehicles caused significant expression of inflammation genes [47].

Thus, while MSC-EVs act as broad-spectrum modulators of inflammation, apoptosis, and regeneration through a generic, “off-the-shelf” set of signals, organ-specific EVs function as precision tools, delivering cargo fine-tuned by evolution to the unique physiological and regenerative needs of their tissue of origin. This is a difference not merely in degree but in kind. For example, the heart-specific miRNA signature of cardiomyocyte-derived EVs directly targets pathways central to cardiac contractility, electrophysiology, and energy metabolism—processes largely irrelevant to the cargo of MSC-EVs. The differences described above (and in the next sections) translate into functional advantages: organ-specific EVs demonstrate greater efficacy, superior targeting in some cases, reduced off-target effects, and a more favorable safety profile compared to their MSC-derived counterparts. This conceptual framework, that organ-specific EVs are not just another source of EVs but represent a qualitatively different therapeutic modality, extends beyond the heart to other organ systems discussed in this review.

## 3. Brain-Derived EVs

Acute ischemic stroke represents another socially significant pathology, being a leading cause of permanent disability and mortality worldwide, which underscores the critical need for novel, effective strategies for neuroprotection and recovery. Key therapeutic targets following cerebral ischemia include neuronal cell death, disruption of the blood–brain barrier (BBB), and the activation of neuroinflammatory cascades that exacerbate tissue damage. Currently, significant attention is focused on EVs derived from MSCs as a promising neuroprotective approach due to their regenerative and anti-inflammatory potential. However, as universal modulators, MSC-EVs may lack the specificity required for targeted repair of complex neuronal circuits. This limitation has led to exploring more specialized EV sources, such as neural progenitor cells and, ultimately, differentiated brain cells, including astrocytes, microglia, and endothelial cells. It is hypothesized that EVs carrying unique molecular cargo from their donor cells may enable more precise modulation of injury mechanisms, thereby opening new horizons for stroke therapy.

### 3.1. Neural Progenitor Cells-Derived EVs

Much attention is paid to the use of EVs isolated from neural progenitor cells (NPC-EVs) as a promising tool for modulating the BBB and reducing the inflammatory response in stroke. During I/R, activation of NF-κB occurs in the brain, associated with increased secretion of pro-inflammatory cytokines such as TNF-α, IL-1β and IL-6. Studies have shown that exosomes from NPCs (NPC-exosomes) effectively suppress NF-κB activation and reduce the levels of these cytokines in microglial cells (BV2) [48]. Thus, NPC-exosomes reduced microglial activation, as shown by Iba1 staining, suggesting their neuroprotective effects through modulation of neuroinflammation [48]. Indeed, in the middle cerebral artery occlusion (MCAO) mouse model, NPC-EVs significantly reduced leukocyte infiltration into the brain and altered the immune response profile. The relative proportion of T- and B-lymphocytes increased, which may indicate a more controlled recovery after stroke [49].

NPC-EVs also reduced the expression of MMP-9, an enzyme that degrades the extracellular matrix and increases BBB permeability [49]. EVs were shown to promote increased activity of the ATP-binding cassette transporter B1 (ABCB1, also known as P-gp), which is important for maintaining BBB functionality [49]. All these factors indicate a dual mechanism of action of NPC-EVs—anti-inflammatory and BBB-protective.

Analysis of NPC-exosomes revealed the presence of specific miRNAs, including let-7g-5p, miR-99a-5p, let-7i-5p, miR-139-5p, miR-98-5p, miR-21-5p, and let-7b-5p. These miRNAs regulate MAPK and NF-κB signaling pathways, thereby suppressing inflammation and protecting the BBB [48]. RT-PCR confirmed the high expression of these miRNAs in NPC-exosomes and their involvement in the modulation of the pro-inflammatory pathways in BV2 cells. In the MCAO model, administration of NPC-exosomes significantly reduced infarct volume, improved neurological parameters, and protected the BBB from damage, as demonstrated by permeability to both small molecules of the dye Lucifer Yellow and large complexes such as Evans Blue-Albumin [48]. This data underlines a complex neuroprotective effect of NPC-exosomes, including antioxidant, anti-inflammatory and barrier-saving activities.

### 3.2. Astrocyte- and Microglia-Derived EVs

Activated astrocytes release exosomes that promote axon growth and restore neuroplasticity. Modulation of the Sema3A (semaphorin 3A)/Rnd1 (Rho family GTPase 1)/R-Ras/Akt/GSK-3β (glycogen synthase kinase-3β) signaling pathway in rats subjected to MCAO results in an increase in the regenerative activity of these exosomes. In particular, the presence of prostaglandin D2 synthase in astrocyte exosomes is associated with improved axonal recovery. Sema3A inhibition enhances astrocytic exosome efficiency and improves functional recovery after stroke [50].

M2-phenotype microglia also secrete exosomes (BV2-Exo), which demonstrate a pronounced neuroprotective effect in ischemic brain injury. These exosomes reduce neuronal apoptosis and infarct volume and improve neurological functions in a mouse model of transient MCAO. Transfer of miRNA-124 and miRNA-137 into neurons is described as one of the main mechanisms of action. Thus, miR-124 reduces the level of ubiquitin-specific protease 14, which promotes neuronal survival, and miR-137 interacts with the Notch1 pathway, providing protection of neurons from damage. Inhibition of these miRNAs was shown to significantly attenuate the neuroprotective effects of BV2-Exo [51,52].

### 3.3. Endothelial Cells-Derived EVs

Similarly to heart diseases, endothelial cell-derived EVs play an important role in neuronal regeneration and restoration of brain function after ischemic stroke. Recent studies highlight their therapeutic potential, especially through the transfer of specific miRNAs such as miR-126 and miR-124 and modulation of neuroplasticity. Experiments on ischemic stroke models in rats (MCAO/R) have shown that administration of exosomes isolated from brain endothelial cells (bEnd.3) improves motor function and promotes restoration of synaptic transmission and plasticity in the cerebral cortex. The exosome-treated group also showed an increase in dendritic length and improved neuritogenesis compared to the control group. The key role in stimulation of neurite growth is attributed to the content of miRNA-126-3p in endothelial exosomes [53].

Endothelial exosomes promote angiogenesis and vascular remodeling after ischemia by improving the survival and angiogenic activity of ischemia-injured bEnd.3 via miRNA-126 signaling [54]. This promotes the restoration of microcirculation, improvement of perfusion of the ischemic zone, and reduction in infarction volume. MiRNA-126 regulates the expression of genes associated with angiogenesis and vascular remodeling, including VEGFR2 (vascular endothelial growth factor receptor-2), CD147, and the MMP pathway.

One of the important therapeutic effects of endothelial EVs is the maintenance of the integrity of the BBB during ischemia. Exosomes have been shown to suppress Cav-1 activity, resulting in the reduction in BBB damage and its recovery [55], the reduction in leukocyte infiltration, and the prevention of cerebral edema. This is mediated by suppressing signaling pathways involved in barrier destruction, such as Cav-1/CD147/VEGFR2/MMP [55]. Separately, inhibition of acid sphingomyelinase and reduction in ceramide levels have been shown to enhance exosome production by endothelial cells, which also promotes angiogenesis and brain remodeling after stroke [52]. Furthermore, EVs can reduce neuroinflammation by reducing the activity of microglia and astrocytes. For example, exosomes containing miRNA-124 inhibit the STAT3 signaling pathway and reduce gliosis and the inflammatory response in the ischemic area [56].

However, miR-155, contained in the exosomes of endothelial cells, has been shown to inhibit the proliferation and migration of endothelial cells, increasing vascular permeability [53]. This highlights the importance of regulating the miRNA content in vesicles to achieve a therapeutic effect.

## 4. Kidney-Derived EVs

I/R injury is the main cause of AKI. I/R can affect any part of the nephron, but proximal tubular epithelial cells (TECs) are particularly sensitive to hypoxia, as they are rich in mitochondria and have a high metabolic rate. Proximal TECs damage caused by ischemia plays a key role in the development of AKI and its transition to chronic kidney disease [57]. A large amount of research in the field of regenerative medicine has described therapeutic effects of vesicles produced by MSCs. However, it remains unclear whether vesicles produced by TECs have any effect on kidney cells during injury. Recent studies suggest a key role for renal cell EVs in regulating pathological processes associated with I/R renal injury. EVs produced by proximal TECs, podocytes, and endothelial cells from renal microvasculature are described.

### 4.1. EVs Produced by Proximal TECs

Below, we will provide evidence that vesicles produced by proximal TECs under normal conditions alleviate ischemic renal injury when administered intravenously, and vesicles produced under pathological conditions have an even more significant effect on post-ischemic recovery.

Intravenous administration of exosomes produced by TECs under hypoxic conditions improved renal function in many ways: decreased levels of oxidative stress, fibrosis, and inflammation and reduced loss of peritubular capillaries and glomeruli [58,59]. The protective effect of exosomes produced by damaged cells may be provided by miRNA delivery [60]. In particular, miR-20a-5p, enriched in exosomes from damaged tubular cells, can inhibit mitochondrial damage and apoptosis of TECs in I/R-induced AKI both in vitro and in vivo [61]. The beneficial effect of hypoxic proximal renal tubule exosomes on renal cell apoptosis could be mediated by HIF-1 (hypoxia-inducible factor-1): exosomes from hypoxia-exposed cells significantly suppress apoptosis in vitro, while exosomes from hypoxic cells with HIF-1 knockdown have no effect on apoptosis [62]. However, it is still hard to say the protective effect of hypoxic exosomes completely depends on HIF-1 because numerous transcription factors and cellular signaling pathways are activated during hypoxia that may mediate the protective effect. Exosomes can also regulate autophagy by delivering miR-590-3p to recipient cells in vitro; this miRNA enhances autophagy and inhibits apoptosis, thus protecting kidney cells from I/R injury [63]. Intercellular communication during renal I/R injury may also be mediated by mRNA in exosomes. In particular, mRNA of ATF3 (activating transcription factor 3), the level of which is increased in TEC-derived exosomes after I/R, inhibits the expression of the pro-inflammatory MCP-1 gene and alleviates I/R-induced AKI [64]. The protein cargo of exosomes may also play a role in renal recovery after I/R injury. For example, exosomes produced by hypoxic TECs have increased levels of VEGF-A, which promotes the proliferation of peritubular endothelial cells and thus prevents progression of AKI [65].

However, exosomes may not only mediate recovery processes but also play a role in the mechanisms that promote disease progression, which should be taken into account when using them as a therapy. Thus, miRNA of exosomes produced by hypoxic TEC can induce ferroptosis of renal tubular cells [57]. In addition, exosomes produced by hypoxic TEC can be taken up by macrophages and induce their polarization into the M1 type by the transfer of miR-374b-5p, miR-106b-5p, and miR-23a, promoting tubulointerstitial inflammation and aggravating AKI [66,67,68]. Exosomes produced by renal TECs under hypoxic conditions can also activate fibroblasts and worsen renal fibrosis by transferring miR-150-5p and TGF-β1 (transforming growth factor beta 1) mRNA [69,70,71]. It should be noted that biodistribution in the body (see Section 9.1) and preservation of the functional activity of exosomes must be carefully tested in animal models of I/R renal injury in vivo.

### 4.2. EVs Produced by Podocytes

Podocyte-derived vesicles have been reported to activate inflammatory and fibrotic processes, as well as to aggravate the development of glomerular damage and sclerosis. Podocyte microvesicles are known to be taken up by renal TECs and promote fibrotic changes through phosphorylation of p38 MAPK and activation of a further signaling cascade [72].

Exosomes produced by angiotensin II-damaged podocytes exacerbate proteinuria and enhance the loss of ZO-1 and podocalyxin, which disrupts the integrity of podocyte gap junctions and leads to further damage to the filtration barrier. Exosomes also act as mediators between podocytes and mesangial cells, transmitting signaling molecules, such as Sonic Hedgehog signaling protein, which activates mesangial cells. Podocyte exosomes increase inflammation and fibrosis in the glomerulus, linking proteinuria with the development of glomerulosclerosis: activation of mesangial cells leads to increased synthesis of extracellular matrix (e.g., fibronectin, α-SMA) [73]. Exosomes may also act as platforms for antigen presentation, activating T-cells and promoting their infiltration into the glomeruli, which enhances the inflammatory response and tissue damage.

Modulation of the release of cargo from podocyte exosomes may be a promising therapeutic strategy to prevent the progression of chronic kidney disease. Blocking Sonic Hedgehog signaling (e.g., with a cyclopamine inhibitor) or inhibiting EVs biogenesis (e.g., with dimethyl amiloride) prevents mesangial cell activation and slows the progression of sclerosis [73].

### 4.3. Vesicles in Urine

Urine contains a heterogeneous population of EVs originating from various parts of the urogenital tract, including the kidneys [74]. Urine seems to be a more promising source of EVs for therapeutic use than culture medium, since larger amounts of vesicles can be isolated from urine [75].

The large number of exosomes in urine makes it easy to isolate them, label them with fluorescent dyes, and subsequently track them throughout the body [75]. When administered intravenously to healthy mice and mice with AKI, the urinary exosomes were predominantly localized in the damaged kidneys, as shown by optical imaging. They accelerated renal recovery by reducing functional and histological impairments and stimulating tubule cell proliferation. Moreover, the introduction of urinary exosomes dramatically suppressed the activation of pro-inflammatory cytokines during injury. Klotho factor, a transmembrane protein crucial for the regeneration of renal tissue, was identified in the exosomes, which ensured the restoration of Klotho levels in the damaged tissue after the introduction of urinary exosomes [75]. Urinary exosomes can alter the expression of renal tubular target genes through the delivery of miRNAs: the most abundant miRNAs are members of the miR-10, miR-30, and let-7 families [76]. Some of them (miR-30 and miR-151) were proven to be transferred to injured renal tissue [75].

Exosomes with specific molecular profiles obtained from urine samples may become promising non-invasive biomarkers of kidney diseases and complement conventional invasive methods, including kidney biopsy.

Thus, kidney-derived exosomes may both reduce the spread and progression of I/R injury to renal cells and promote damage and death of renal epithelial cells. The dual effect of exosomes on recipient cells is determined by a complex regulatory network of miRNA, mRNA, and the proteins of which they are composed. This requires a systematic approach to study the mechanisms underlying the effects of renal EVs.

## 5. Liver-Derived EVs

I/R injury of the liver most commonly occurs during transplantation and leads to early graft failure, dysfunction, and rejection. Currently, the role of EVs in the liver under conditions of injury is poorly studied; however, their function in the regulation of biological processes is being actively investigated. Recent studies are focused on the functional effects of EVs derived from various liver cell types, particularly hepatocytes and stem cells.

It has been shown that exosomes derived from hepatocytes deliver neutral ceramidase and sphingosine kinase 2 to hepatocytes for the synthesis of sphingosine-1-phosphate, which appears to play a key role in proliferation and regeneration [16]. Interestingly, in contrast to the dose-dependent enhancement of proliferation induced by hepatocyte-derived exosomes, exosomes from sinusoidal endothelial cells did not promote proliferation, while Kupffer cell exosomes even led to its reduction [16]. Furthermore, hepatocyte-derived vesicles generated upon Yes-associated protein activation under injury conditions carry the CD47 receptor on their surface. This receptor suppresses dendritic cell activation and contributes to protection against liver I/R injury [77]. Thus, CD47-enriched EVs could represent a promising therapeutic approach for liver ischemic–reperfusion injury [77].

However, the role of hepatocyte-derived EVs is ambiguous, as IRF-2 (interferon regulatory factor 2)-Rab27a (Ras-related protein)-regulated EVs activate the TLR4 (Toll-like receptor 4) pathway in neutrophils, promoting injury development [78]. It has also been shown that exosomal miR-122-5p contributes to acute liver inflammation following I/R by inducing the polarization of Kupffer cells into M1-phenotype through the suppression of PPARδ (peroxisome proliferator-activated receptor delta) and activation of NF-κB [79]. The data suggest that inhibition of Rab27 or reduction in circulating miR-122-5p could be considered a potential therapeutic approach for treating the liver after injury [78,79].

For the therapy of liver I/R injury, the application of vesicles derived from liver stem cells is also being considered. An administration of vesicles from human liver stem cells to mice after ischemic injury significantly reduced the levels of ALT (alanine aminotransferase) and LDH (lactate dehydrogenase), and decreased necrosis and the expression of cytokines TNF-α, CCL-2 (C-C motif chemokine ligand 2), and CXCL-10 (C-X-C motif chemokine ligand 10), thereby exerting a hepatoprotective effect [80]. In a model of warm hepatic ischemia in rats, human liver stem cell vesicles not only contributed to a reduction in ALT and AST (aspartate aminotransferase) levels by improving liver metabolism through phosphate uptake and pH regulation but also reduced necrosis and enhanced proliferation [81].

Thus, vesicles derived from either hepatocytes or liver stem cells represent multifunctional agents for the therapy of liver ischemic injury. Nevertheless, they can also contribute to both the improvement and worsening of liver tissue condition, depending on a number of factors.

## 6. Intestine-Derived EVs

Intestinal injury caused by I/R involves all aspects of the intestinal mucosa’s mechanical, immune, and microbial barriers [82]. Intestinal ischemia leads to oxidative stress, activation of the inflammatory response, intestinal dysfunction, and even multiple organ failure [83]. Under these conditions, EVs isolated from intestinal epithelial cells can exert either damaging or protective effects depending on their molecular content and origin [84]. EVs can be released from intestinal epithelial cells, endothelial cells, and immune cells in both normal conditions and during intestinal I/R.

EVs, released by intestinal epithelial cells, play a crucial role in regulating inflammatory processes, both in focal injury and in systemic pathology such as sepsis. These vesicles contain a wide range of signaling molecules—miRNAs, circular RNAs (circRNAs), proteins, and cytokines—that are involved in modulating immune responses, maintaining barrier integrity, and promoting tissue repair [85]. MiRNAs cargo (e.g., miR-210, miR-155, miR-23a-3p) can regulate apoptosis and tight junction integrity. On the contrary, during ischemia, stressed or dying intestinal cells release EVs containing DAMPs (damage-associated molecular patterns), ROS, and pro-inflammatory cytokines. These EVs can activate local immune cells (e.g., macrophages, neutrophils), exacerbating inflammation and tissue injury [86].

The non-coding circRNA circEZH2_005 was identified in exosomes derived from intestinal crypt stem cells. The levels of this molecule decrease during I/R, both in intestinal tissues and in circulating blood exosomes [87]. Increased expression of circEZH2_005 in vivo reduces apoptosis, suppresses inflammation, and promotes the proliferation of intestinal stem cells and the restoration of the epithelial barrier. The molecular mechanism of circEZH2_005 effects based on interaction with hnRNPA1 and modulation of the Hippo signaling pathway via Gprc5a [87].

MiRNA miR-23a-3p was also found in exosomes secreted by the intestinal epithelium. During I/R, miR-23a-3p downregulates MAP4K4 (mitogen-activated protein kinase 4), a key regulator of the inflammatory response, thereby reducing oxidative stress and the production of pro-inflammatory cytokines such as TNF-α and IL-6 [88]. This highlights the importance of the miR-23a-3p/MAP4K4 axis in protecting against I/R-induced injury.

Impaired intestinal barrier function leads to bacterial translocation and activation of the systemic inflammatory response and sepsis. In this condition, EVs demonstrated both anti-inflammatory and pro-inflammatory effects. EVs isolated from the intestinal lumen during sepsis contain anti-inflammatory miRNAs that suppress the expression of pro-inflammatory cytokines such as TNF-α and IL-17A in the intestinal mucosa [85]. EVs, containing TGF-β1, induce the maturation of regulatory T-cells and dendritic cells, contributing to the restoration of immune homeostasis [89]. However, some types of EVs exacerbate inflammation, especially when contained with pro-inflammatory cytokines (IL-6, IL-1β, IL-22), pro-inflammatory miRNAs, DAMPs, or bacterial components [85]. Their production increases upon activation of TLRs and other pathogen-recognition pathways, especially in sepsis.

Several proteins and molecules play a key role in the targeting and functional activity of gut EVs: EpCAM (epithelial adhesion molecular antigen) ensures the specific localization of EVs in the intestine. In its absence, EVs lose tissue specificity and fail to protect the gut from inflammation [90]. Inflammatory bowel diseases can activate the ERK pathway and enhance TGF-β1 production in EVs, enhancing their immunoregulatory properties. Delivery of EVs enriched with anti-inflammatory molecules (e.g., TGF-β1, miR-23a-3p, circEZH2_005) could become a novel approach in treating acute inflammatory conditions. On the other hand, analysis of the composition of EVs allows for predicting outcomes in sepsis and assessing the extent of intestinal damage without invasive methods. For instance, levels of circEZH2_005, miR-23a-3p, and other non-coding RNAs in plasma EVs can serve as markers of early intestinal injury in I/R or sepsis. Thus, EVs represent a powerful mechanism of intercellular communication that plays a role in both the development and limitation of inflammatory reactions during intestinal ischemia and sepsis. Their unique composition and ability to deliver functional molecules make them promising candidates for diagnosis and therapy in gastrointestinal inflammatory diseases and systemic inflammatory conditions.

## 7. Skeletal-Muscle-Derived EVs

Critical limb ischemia (CLI) is the most severe consequence of peripheral artery disease caused by atherosclerotic occlusion of the leg arteries [91]. Although surgical or endovascular interventions are still major therapies for improving blood flow, most patients complain of persistent or recurring symptoms even after successful revascularization [92]. The formation of new capillary blood vessels, neovascularization, is a crucial event in tissue salvage after ischemia. This implies that new therapeutic options for angiogenesis in patients suffering from these symptoms are a major unmet need.

Several studies have shown that stem cells and progenitor cells such as endothelial progenitor cells (EPCs) contribute to neoangiogenesis during hindlimb ischemia, and the mechanisms may be based on paracrine effect, which is realized through the release of cytokines, chemokines, growth factors, and EVs [93]. Systemic administration of EPC-EVs was shown to significantly improve neovascularization and reduce the extent of muscle damage in mice with a model of acute hindlimb ischemia. It was found that EPC-EVs enhance perfusion, increase capillary density, stimulate the regeneration of muscle fibers, and reduce necrosis. The inactivation of EV RNA content by ribonuclease or DICER (large multidomain endoribonuclease that plays a central role in the miRNA and RNA interference pathways) treatment reduces the therapeutic effect, highlighting the importance of RNA in the mechanism of action of these vesicles.

It was shown that microvesicles isolated from ischemic muscle tissue are capable of activating EPC, enhancing their proliferation and migration, and inducing the expression of VEGFR-2 and activation of the Akt pathway [94]. These effects lead to enhanced postnatal vasculogenesis and improved blood flow recovery in limb ischemia models in animals. Thus, EVs derived directly from damaged tissue or from EPC act as mediators of intercellular communication, transferring signaling molecules to ischemic tissues, and play an important role in initiating and coordinating endogenous regenerative processes.

Serum EVs also demonstrated pronounced angiogenic activity in a mouse model of acute hindlimb ischemia: they promoted vascular network remodeling and prevented skeletal muscle damage [95]. The main mechanism of serum EV action is the activation of the TGFβ1/ALK1 (anaplastic lymphoma kinase 1)/SMAD1–5/ID1 (inhibitor of DNA-binding 1) signaling pathway, which enhances endothelial cell proliferation and the formation of new capillaries. In addition, such EVs modulate the MMPs and cytokines, creating a favorable microenvironment for angiogenesis and tissue regeneration.

Some studies suggest modifying EVs to enhance their therapeutic activity for the treatment of CLI. For example, the study by Xing et al. [96] describes the use of engineered EVs obtained from endothelial cells overexpressing VEGF and TFEB (transcription factor EB) and their delivery via a hydrogel. The authors showed a significant improvement in perfusion recovery in mice after local application of the EV hydrogel, reduced inflammatory infiltration (decreased number of CD68+ macrophages), and enhanced angiogenesis and muscle tissue regeneration through dual activation of angiogenic and metabolic pathways. This approach opens up new possibilities for the development of bioengineered therapeutic agents based on EVs, aimed at increasing their specificity and efficacy in ischemic diseases.

## 8. Lung-Derived EVs

Although the lungs usually do not experience ischemia, acute lung injury (ALI) and acute respiratory distress syndrome (ARDS) are common pathological conditions that most often occur due to sepsis, trauma, or massive blood loss [97]. To date, few effective pharmacological therapies has been developed, resulting in ALI having a high mortality rate [98]. EVs derived from lung cells, particularly alveolar epithelial and pulmonary endothelial cells, facilitate communication between alveolar cells, and their application in the therapy of ALI is being actively investigated [99,100].

The first line of defense for the alveolar–capillary barrier is the lung epithelial cells, and their vesicles play a significant role in regulating the functions of lung macrophages [97]. It is known that during sepsis, alveolar epithelial cells secrete exosomes containing the miRNAs miR-92a-3p and miR-146a, which lead to the activation of the NF-κB pathway in alveolar macrophages, thereby triggering an inflammatory response [101,102]. Conversely, alveolar epithelial cell exosomes containing miR-1249-5p and miR-27b-3p can suppress alveolar macrophage function and the development of the inflammatory response by inhibiting the NF-κB signaling pathway, which could be effective for the therapy of ALI [103,104]. Indeed, administration of EVs from bronchial epithelial cells mitigated ALI by reducing inflammatory cell infiltration and attenuating fibrosis through miRNA-mediated inhibition of NF-κB and the interaction between TGF-β and Wnt signaling pathways [105,106].

The severity of lung injury depends on the integrity of the pulmonary endothelial barrier. Similarly to alveolar epithelial cells, endothelial cells regulate alveolar macrophage function through the secretion of exosomes, leading to enhanced anti-inflammatory and anti-fibrotic effects [104]. One of the promising approaches for the therapy of ALI is the use of engineered EVs of endothelial origin, LET-EVs-miR-125b. LET-EVs-miR-125b suppressed apoptosis, restored intercellular contacts, and stimulated proliferation by delivering miR-125b to lung endothelial cells, thereby providing protection for the lung endothelial barrier in vivo and in vitro in ARDS [107].

During ALI, exosomes produced by M1 macrophages promote ferroptosis and exacerbate damage due to miRNA cargo [108,109,110]. Exosomes derived from M2 macrophages effectively suppressed the pyroptosis of alveolar macrophages and prevented the excessive release of cytokines both in vivo and in vitro via miR-709 by the inhibition of the NF-κB/NLRP3 signaling pathway [111].

Thus, the secretion of EVs by alveolar and bronchial epithelial cells, endothelial cells, and macrophages plays a crucial role in cellular communication under both normal conditions and lung injury. However, since native EVs are unable to exert a sustained effect on damaged lung tissue and carry only a limited amount of bioactive cargo, artificially modified EVs represent a platform for the development of therapeutic approaches in lung injury.

## 9. Challenges and Future Directions of Therapy Mediated by Organ-Specific EVs

### 9.1. Biodistribution of Organ-Specific EVs 

A critical and often underappreciated aspect of organ-specific EV therapy is their in vivo biodistribution, particularly following systemic administration. While the concept of “organ-specific tropism” is intuitively appealing—suggesting that EVs derived from a given tissue preferentially home to that same tissue—experimental evidence supporting this notion remains limited and, in several key cases, contradictory. This issue is especially relevant in the context of I/R injury, where disruption of biological barriers (e.g., endothelial integrity, blood–brain barrier) and systemic inflammation may significantly alter EVs trafficking. However, even in healthy animals, the dominant pattern of EVs biodistribution after intravenous injection is non-specific accumulation in filtering organs, primarily the liver and spleen, followed by the lungs—consistent with the behavior of most nanoparticles of similar size.

The liver is the primary organ where EVs localize, with peak detection observed both within the first hour and during the period from 2 to 12 h. The lungs and spleen are other organs capable of accumulating EVs [112]. Liver-derived EVs exhibit pronounced tropism for the liver upon systemic administration. Studies have shown that exosomes derived from liver stem cells predominantly accumulate in the liver (especially under fibrotic and inflammatory conditions), with minor accumulation in the lungs, while no signal is detected in the spleen and kidneys [112,113,114]. In contrast, EVs isolated from liver progenitor cells and modified by the removal of terminal sialic acid demonstrate increased mobility, more efficient cellular interaction, and enhanced uptake capacity, which elevate their accumulation in the lungs instead of the liver [114]. In the liver, hepatocyte-derived EVs carrying CD47 can suppress dendritic cell activation [77], but again, no comparative biodistribution data exist to demonstrate preferential liver uptake over other reticuloendothelial organs. Biodistribution also depends on the administration route. While intravenous injection is less effective, delivery via the portal vein results in the fastest and highest accumulation of EVs in the liver [115]. The highest lung delivery of EVs is achieved through intratracheal or inhalation administration, in contrast to intravenous injection, where a portion of the vesicles is diverted to the liver and spleen [116,117].

For heart-derived EVs, CPC-EVs are frequently cited as an example of organ-specific targeting due to their ability to deliver therapeutic cargo (e.g., VEGF-A mRNA) to the heart more efficiently than synthetic nanoparticles or MSC-EVs [47]. However, this conclusion is based on functional readouts and local protein expression, not on direct comparative biodistribution data across multiple organs. In fact, multiple independent studies directly tracking fluorescently or radiolabeled CPC-EVs or cardiosphere-derived EVs after intravenous injection in healthy rodents consistently report minimal cardiac accumulation. For instance, 90 min after intravenous injection, CPC-EVs showed a typical nanoparticle-like biodistribution pattern, with CPC-EVs mainly localized in the liver and rarely in the heart [118]. In a previous report, healthy mice received retro-orbital injections of cardiosphere-derived exosomes, and 2 h post-systemic injection, they accumulated primarily in the lung, spleen, and liver, with nearly undetectable levels in the heart [119]. Several studies reported similar results, with CPC-EVs and cardiosphere-derived EVs accumulating primarily in the liver after intravenous injection in healthy mice [120,121]. These findings strongly suggest that cardiac EVs do not possess an intrinsic, active homing mechanism to the heart under physiological conditions. The apparent “cardiac specificity” observed in therapeutic studies may instead reflect enhanced retention or functional uptake in injured tissue or superior cargo bioactivity once EVs reach the heart (even at low levels), rather than superior delivery.

For brain-derived EVs, systemic delivery faces the formidable obstacle of the intact BBB. While neural progenitor cell exosomes or brain endothelial EVs show neuroprotective effects in stroke models [48,49,53], direct evidence of their brain accumulation after intravenous injection is scarce. Most likely, their efficacy stems from action on the compromised BBB periphery, modulation of peripheral immune cells (e.g., splenic monocytes [45]), or, in many studies, local (intranasal or intracerebroventricular) administration, which bypasses systemic biodistribution altogether.

For kidney-derived EVs, the situation is slightly more nuanced. Urinary EVs and proximal-tubule-cell-derived EVs have been shown to accumulate in injured kidneys after intravenous injection in AKI models [75]. However, it remains unclear whether this reflects active targeting or passive retention due to enhanced vascular leakiness in the post-ischemic kidney and filtration mechanisms inherent to renal physiology. Crucially, no study has quantitatively compared kidney-EV accumulation in the kidney versus the liver or spleen in the same animal, making it impossible to claim true organ specificity.

Future studies must include rigorous, multi-organ quantitative biodistribution analyses (e.g., using radiolabels, luciferase reporters, or mass spectrometry-based tracking) in both healthy and I/R-injured models to definitively address the organotropism question. Until then, claims of “natural targeting” should be tempered with caution.

### 9.2. Isolation Challenges and Scalable Production Technologies

There are some limitations for organ-specific EVs isolation. Exosomes are present in low concentrations in most biofluids, and many isolation methods achieve poor recovery. Contaminants coexist with EVs, such as large apoptotic bodies, lipoproteins, protein aggregates, and nucleic acid complexes in biological fluids, which can compromise downstream applications [122]. To overcome isolation limitations and meet clinical demand, scalable production strategies focus on both enhancing exosome yield from source cells and achieving efficient, high-purity isolation.

Ultracentrifugation has been the “gold standard” for EV isolation for decades [123]. However, it has serious drawbacks: high g-forces cause co-precipitation of non-vesicular material [124,125,126] and can damage EVs, leading to aggregation, loss of integrity, or lysis—issues that are particularly problematic for therapeutic use. Thus, ultracentrifugation appears to be a low-specificity method that can potentially damage the target product.

One of the most promising strategies is the combination of size-exclusion chromatography (SEC) and ultracentrifugation [127]. SEC effectively separates vesicles from free proteins, while subsequent ultracentrifugation allows concentration of the purified fraction. This tandem approach significantly improves sample purity and preserves membrane integrity. However, SEC has its own limitations as it requires large sample volumes and is not easily scalable [128].

Another powerful tool is the tangential flow filtration (TFF) technique, which enables efficient isolation of EVs from large volumes of biological fluids, such as lipoaspirate from patient samples, or when scaling up EV production from cell cultures. It is a scalable and sterile method for obtaining EV-enriched samples that can be used clinically [129,130]. Another method, asymmetric flow field-flow fractionation (AF4), is a gentle, highly reproducible technique that enables efficient separation of nanoparticle subpopulations [131]. Using this method it is possible to effectively separate and analyze nanoparticles of different sizes, which have been shown to be enriched in various protein and lipid markers and have different biodistribution patterns in organs [132].

The bottleneck of the field is the large-scale production of purified EVs for clinical applications. Conventional 2D cultures yield insufficient EV quantities, prompting a shift toward 3D culture systems and bioreactors (e.g., hollow-fiber or microcarrier-based), which can increase EV production by 100–140-fold while better preserving native cell phenotypes [122]. In bioreactors with precisely controlled conditions (pH, oxygen, nutrients), cells secrete 5–10 times more EVs. Moreover, 3D cultures that mimic the tissue microenvironment not only boost EV production but also alter EV profiles, making them more “physiologically relevant”. However, a major challenge for organ-specific EVs is the limited availability and poor expandability of primary cells (e.g., cardiomyocytes, neurons, renal tubular cells). To overcome this, researchers are turning to engineered, non-tumorigenic cell lines—such as iPSC-derived progenitors or immortalized MSCs—as stable, standardized, and scalable sources of organ-tropic EVs.

Perhaps the most disruptive approach is the shift from harvesting naturally secreted EVs to the manufacture of EV-mimetic nanovesicles. Techniques like cell extrusion or nitrogen cavitation allow for the production of biological nanoparticles from a small number of cells at yields up to 250-fold higher than natural secretion. These engineered vesicles can be loaded with defined therapeutic cargo and retain the surface properties of their parent cells, offering a highly controllable and scalable alternative that bypasses many of the limitations of traditional EV biomanufacturing.

In conclusion, for clinical translation, we need standardized protocols that are reproducible across laboratories. Therefore, alongside production innovations, we must advance EV characterization methods, which include extensive evaluation of several parameters, including particle quantities, size distribution, morphology, purity, protein content, RNA content, and biological activity.

### 9.3. Clinical Translation: Current Status of EV-Based Trials

There are few registered trials representing early-phase (primarily Phase I/II) clinical studies designed to test the feasibility of using extracellular vesicles as a cell-free therapeutic strategy for burn and wound healing (NCT05078385, NCT05475418, [133]) and very few for acute ischemic and respiratory conditions (NCT04327635, NCT04657458). EV-based therapy is being explored as a cell-free alternative to cell therapy, capable of modulating inflammation and promoting tissue regeneration.

It is crucial to emphasize that there are no registered clinical trials to date that have utilized organ-specific extracellular vesicles. This significant gap in translational research highlights an unmet need for the development and evaluation of EVs derived from specific organs, which may possess unique regenerative and immunomodulatory properties tailored to target tissues. Our review is specifically designed to address this gap by synthesizing current preclinical evidence, underscoring the therapeutic potential of organ-specific EVs, and outlining a roadmap for their future clinical translation.

### 9.4. Concluding Remarks and Future Perspectives

EVs derived from specific sources, such as neurons, endothelial cells, and immune cells, possess significant therapeutic potential for I/R injury. They provide robust tissue protection through multi-faceted mechanisms, including the reduction in inflammation, alleviation of oxidative stress, prevention of apoptosis, and stimulation of regeneration. This opens up promising opportunities for the development of novel, cell-free therapeutic strategies based on EVs, independent of stem cells. Such approaches are particularly valuable in clinical scenarios where the use of stem cells is limited due to ethical concerns, potential immunological complications, and the complexity of standardization and regulation as a drug.

Despite their significant therapeutic potential, the clinical translation of organ-specific EVs faces several challenges. A primary limitation is their isolation and scalable production. In contrast to MSCs, which can be expanded in culture to generate large EV yields, obtaining sufficient quantities of organ-specific EVs is considerably more difficult. Furthermore, the molecular cargo of EVs is highly dependent on the physiological state of the donor cells, which can be influenced by in vitro culture conditions or the in vivo health status of the donor. This variability complicates the production of standardized, clinical-grade EV preparations with consistent therapeutic efficacy, as even subtle changes in culture parameters or the need to pre-condition cells to mimic injury can drastically alter the EVs’ content. Secondly, conventional culture media, often supplemented with fetal bovine serum, introduce significant confounders, including xenogeneic EVs and non-EV factors that are co-isolated with cell-derived EVs, which may lead to misinterpretation of cargo and function. More physiologically relevant culture systems, such as EV-depleted or plasma-like media, may better mimic the native cellular microenvironment and yield EVs with enhanced tissue-specific functionality and reduced stress-induced artifacts [134]. Thus, optimizing culture conditions is not merely a technical detail but a critical determinant of EVs’ therapeutic efficacy and safety. Moreover, the functional outcome of EV administration is context-dependent; depending on the source, cargo, and the timing of administration, EVs can paradoxically mediate both protective and detrimental effects, such as promoting pro-inflammatory or pro-apoptotic signaling.

Future studies must focus on overcoming these obstacles through rigorous standardization and targeting EV content. EVs from different cell types exhibit substantial variability in protein, lipid, and nucleic acid content, which directly influences their biological activity and targeting specificity. The use of EVs as a therapy for I/R injuries of various organs urgently requires standardized protocols for EVs isolation, characterization, and dosing to ensure reproducibility and safety. The high heterogeneity of EVs is not merely a technical inconvenience but a fundamental biological feature that must be addressed through advanced isolation techniques (e.g., affinity-based sorting for specific surface markers), comprehensive multi-omics profiling, and functional validation to ensure safety, reproducibility, and efficacy in treating I/R injury.

Bioengineering approaches for EVs have emerged as powerful strategies to enhance the therapeutic efficacy of organ-specific EVs in I/R injury. Cargo enrichment is primarily achieved through donor cell engineering, where cells from target organs are genetically modified to overexpress therapeutic molecules (miRNAs and proteins) that are selectively packaged into secreted EVs. The most common method is donor cell engineering, where parent cells are genetically modified to overexpress specific therapeutic molecules (e.g., pro-angiogenic miRNA-210 in MSCs), which are then naturally packaged into the secreted EVs, an approach shown to significantly improve cardiac function in a MI model [135]. As a complementary technique, post-isolation loading allows direct incorporation of cargo into pre-formed EVs using physical methods like electroporation for loading silence RNA or sonication for incorporating hydrophobic drugs like curcumin. To overcome the critical bottleneck of endosomal trapping and ensure efficient cytosolic delivery of their therapeutic cargo, EVs can be engineered with fusogenic proteins such as VSV-G (vesicular stomatitis virus G-protein) on their surface, a strategy that markedly enhances functional cargo delivery to the cytoplasm [136]. Furthermore, the creation of hybrid EVs, which fuse natural EV membranes with synthetic liposomes, aims to merge the innate biological functionality and targeting capabilities of natural vesicles with the superior engineering versatility and scalability of synthetic systems. Collectively, these methods (spanning genetic manipulation of producer cells, physical or chemical cargo loading, and advanced membrane engineering) represent a paradigm shift from simply using native EVs to actively designing next-generation, precision-engineered nanotherapeutics.

## Data Availability

Not applicable.
